# miR-146a Attenuates Sepsis-Induced Myocardial Dysfunction by Suppressing IRAK1 and TRAF6 via Targeting ErbB4 Expression

**DOI:** 10.1155/2018/7163057

**Published:** 2018-08-27

**Authors:** Rui An, Jianxin Feng, Cong Xi, Jian Xu, Lijun Sun

**Affiliations:** ^1^Department of Radiology, Xijing Hospital, The Fourth Military Medical University, Xi'an, China; ^2^Department of Interventional Radiology, Baoji City People's Hospital, Baoji, China; ^3^Department of Neurology, Baoji City People's Hospital, Baoji, China

## Abstract

Myocardial dysfunction is a major manifestation of sepsis and closely associated with the increased mortality. MicroRNA-146 is one of the most important microRNAs identified as a potent negative regulator in innate immune and inflammatory responses induced by lipopolysaccharide (LPS). We aimed to identify the role and potential regulatory mechanism of miR-146a in sepsis-induced cardiac dysfunction with the induction of ErbB4 signaling. H9C2 cells were treated with LPS to induce sepsis, and miR-146a overexpression significantly increased the cell viability, reduced the apoptosis and ROS level, and attenuated the release of proinflammatory cytokines including TNF-*α* and IL-1*β*. Levels of ErbB4, p-NF-*κ*B, NF-*κ*B, TRAF6, IRAK1, caspase 3, Bcl-2, and Bax were measured by Western blot. The overexpression of miR-146a significantly increased the ErbB4 expression, decreased the expression of TRAF6, IRAK1, caspase 3, and the phosphorylation level of NF-*κ*B, and also increased the Bcl-2/Bax ratio, suggesting the inhibition of inflammation and apoptosis. The protective effects were all abolished by the use of siErbB4. In conclusion, our results demonstrated that the overexpression of miR-146a mitigates myocardial injury by negatively regulating NF-*κ*B activation and inflammatory cytokine production via targeting ErbB4 in LPS-induced sepsis.

## 1. Introduction

Sepsis, characterized by a systemic inflammatory response to severe infection and progressive organ damage, is the leading cause of death in hospitalized patients worldwide. Severe sepsis and septic shock can trigger damage to numerous organs including brain, heart, kidney, liver, and lung [1]. There is compelling evidence that cardiac dysfunction is a major complication associated with sepsis-induced mortality. Patients with sepsis-induced cardiac dysfunction have nearly quadruple mortality rate (70~90%) compared with patients without septic cardiomyopathy (20%). Since that, it is important to find novel therapeutic agents and methods against the sepsis-induced cardiac depression [2].

MicroRNAs (miRNAs), which are 21 to 23 nucleotide noncoding RNAs that regulate gene expression at the posttranscriptional level, have recently emerged as a novel class of regulators in many biological processes ranging from embryonic development, organogenesis, and human inflammatory diseases [3]. Recent studies have shown that several miRNAs (miR-146a, miR-125, miR-155, and miR-18b) play an important role in innate immune and inflammatory responses [4–6]. Among them, miR-146a was one of the key molecules in oncogenesis and inflammatory responses [7] that captured our attention. miR-146a directly targets IRAK1 and TRAF6 which are the key adapter molecules in the TLR/NF-*κ*B pathway in the model of cardiac ischemia and reperfusion (I/R) injury and monocytic cell-based endotoxin tolerance. However, the role of miR-146a in the sepsis-induced cardiac dysfunction has not been fully investigated.

In this study, we investigated the effect and mechanism of miR-146a in the sepsis-induced cardiac dysfunction in the LPS-treated H9C2 model.

## 2. Materials and Methods

### 2.1. Cell Culture and Reagents

Heart-derived H9C2 myocardial cells were obtained from the American Tissue Culture Collection (ATCC) and cultured with Dulbecco's modified Eagle's medium (DMEM) supplemented with 10% fetal bovine serum, 100 *μ*g/ml penicillin, and 100 *μ*g/ml streptomycin in humidified air (5% CO_2_) at 37°C. Media were refreshed every 2 days and replaced with a minimal essential serum-free medium for 12 h before drug treatment. After 24 h incubation, cells were harvested and extracted for analysis.

Lipopolysaccharide (LPS) was purchased from Sigma (Sigma Chemical Co., St. Louis, Missouri, USA). TRIzol and Lipofectamine 2000 were purchased from Invitrogen (Invitrogen, Carlsbad, CA). miR-146a mimics and negative control were obtained from RiboBio (RiboBio, Guangzhou, China). ErbB4 antibody was purchased from Abcam (Abcam, Cambridge, MA, USA). Antibodies against p-NF-*κ*B, NF-*κ*B, TRAF6, IRAK1, caspase 3, Bcl-2, and Bax were purchased from Cell Signaling Technology (Beverly, MA, USA). The rabbit anti-goat, goat anti-rabbit, and goat anti-mouse secondary antibodies were purchased from Beyotime (Shanghai, China). Terminal deoxynucleotidyl transferase dUTP nick-end labeling (TUNEL) kits were purchased from Roche (Mannheim, Germany).

### 2.2. CCK-8 Assay

Cell proliferation was detected by Cell Counting Kit-8 (CCK-8) (Sigma-Aldrich, St. Louis, MO, USA) assays. Cells were transfected with miR-146a mimics or negative control (NC) using Lipofectamine 2000 by following the manufacturer's instructions. To knockdown the expression of ErbB4, 100 nM ErbB4 small interfering RNA (siRNA) (RiboBio, Guangzhou, China) was transfected for 48 h. Then, LPS (1 *μ*g/ml) was added for the establishment of a sepsis cell model. All H9C2 cells were incubated with 10 *μ*l of CCK-8 tetrazolium salt for 2 hours, and the absorbance was detected by a microplate spectrofluorometer at a 450 nm wavelength. The proliferation experiments were repeated three times with triplicate wells for each condition.

### 2.3. ROS

Treated cells were washed with a serum-free DMEM culture medium and incubated with 5 *μ*M dihydroethidium (DHE, Beyotime, Nantong, China) at 37°C for 30 min. Then, images were observed with a fluorescent inverted microscope (Nikon, Tokyo, Japan).

### 2.4. TUNEL

Terminal deoxynucleotidyl transferase dUTP nick-end labeling (TUNEL) assay was performed to analyze cell apoptosis according to the manufacturer's protocol (Roche, Mannheim, Germany). After different treatments, the cells were washed with PBS, fixed in 4% methanol-free formaldehyde solution in PBS, and then incubated with terminal deoxynucleotidyl transferase and fluorescein-labeled dUTP. The apoptotic cells were analyzed by fluorescence microscopy. Green fluorescence represents the TUNEL-positive cells, and blue fluorescence represents the nuclei. The apoptotic index was calculated by dividing TUNEL-positive cells by the total number of nuclei visualized in the same field.

### 2.5. qPCR

Total RNA was isolated from H9C2 cells using TRIzol reagents. Quantitative real-time PCR (qPCR) was conducted using a 4800 real-time PCR machine (Bio-Rad). miR-146a level was quantified by qPCR using specific TaqMan assays (Applied Biosystems, USA) and TaqMan Universal Master Mix (Applied Biosystems, USA). Specific primers for miR-146a, TNF-*α*, and IL-1*β* were obtained from Applied Biosystems. Data were quantified with the 2(−DDct) relative quantification method that was normalized to the snRU6.

### 2.6. WB

After sonication, the lysates of cells were centrifuged and the proteins were separated by SDS-PAGE and then transferred to Immobilon-NC membranes (Millipore, Boston, MA, USA). After 2 h 5% skim milk blockage with Tris-buffered saline at room temperature, the membrane was incubated with primary antibodies against ErbB4, p-NF-*κ*B, NF-*κ*B, TRAF6, IRAK1, caspase 3, Bcl-2, Bax, and *β*-actin overnight at 4°C. Then, membranes were incubated with secondary antibodies conjugated with horseradish peroxidase for 1 h at 37°C. Blots were imaged using a Bio-Rad imaging system (Bio-Rad, Hercules, CA, USA) and quantified using the Quantity One software package (West Berkeley, CA, USA).

### 2.7. Statistics

SPSS 18.0 was used to analyze the data which are presented as the mean ± standard error of the mean (SEM). Comparisons among multiple groups were assessed by one-way analysis of variance (ANOVA). The LSD *t*-test was used to make intergroup comparisons. Probabilities of 0.05 or less were considered statistically significant.

## 3. Results

### 3.1. LPS Induces Oxidative Injury and miR146a Upregulation in H9C2 Cells

**Figure 1:**
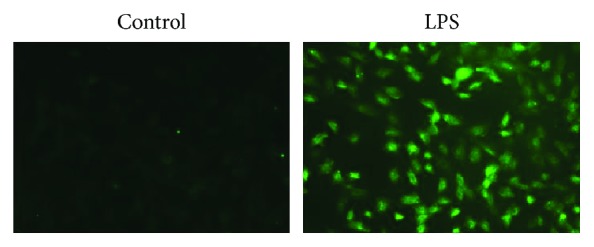
ROS level detected by DHE assay. LPS significantly increased the ROS level.

**Figure 2:**
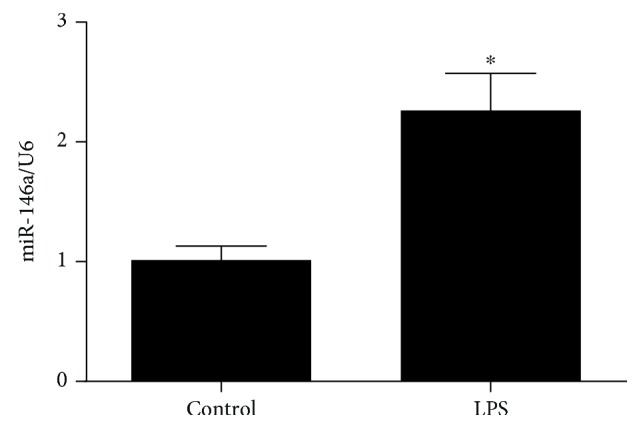
LPS increased miR-146a expression in cardiomyocytes detected by qPCR. ^∗^*P* < 0.05 versus the control group.

**Figure 3:**
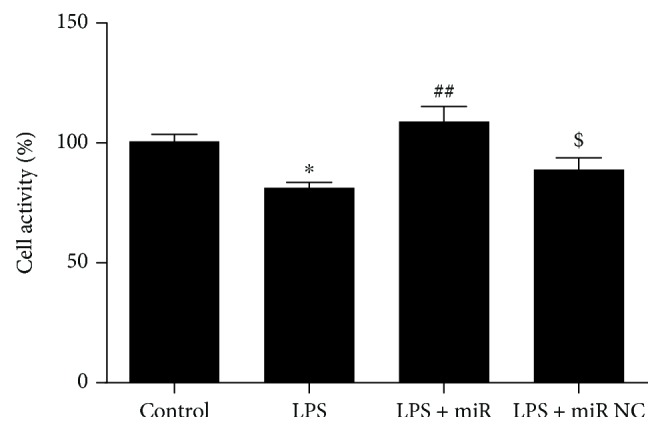
The change of cell viability detected by CCK8. Data were expressed as mean ± SEM. ^∗^*P* < 0.05 versus the control group; ^##^*P* < 0.01 versus the LPS group; ^$^*P* < 0.05 versus the LPS + miR group.

**Figure 4:**
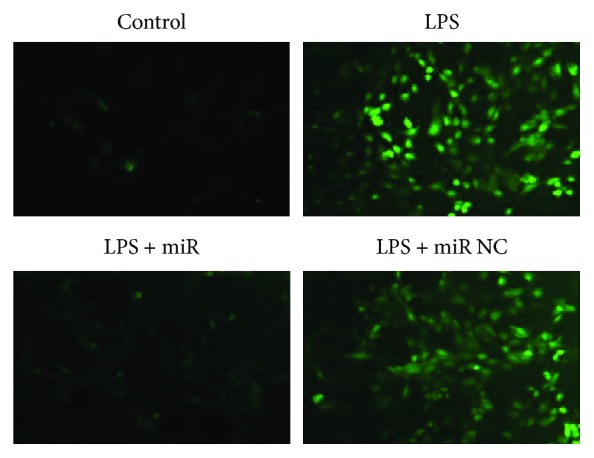
ROS level detected by DHE assay. Overexpression of miR-146a significantly reduced the ROS level post-LPS treatment.

We first examined the oxidative status of cells treated with LPS and the expression of miR-146a. LPS treatment significantly decreased the cell viability measured by CCK8 assay. ROS level was increased in H9C2 cells stimulating with LPS compared with the control group detected by DHE assay (shown in Figure 1). In addition, we measured the miR-146a level in H9C2 cells and the significant endogenous upregulation was detected in the LPS group (shown in Figure 2). Moreover, to explore the role of miR-146a in LPS-induced oxidative injury, we increased miR-146a expression by miR-146a mimics and a negative control (NC) in LPS-treated H9C2 cells. Cell viability was significantly increased, and the ROS production was decreased in the miR-146a group compared with the LPS group (shown in Figures 3 and 4).

### 3.2. Overexpression of miR-146a Downregulated TRAF6 and IRAK1 Expression in Cardiomyocytes following LPS Treatment

**Figure 5:**
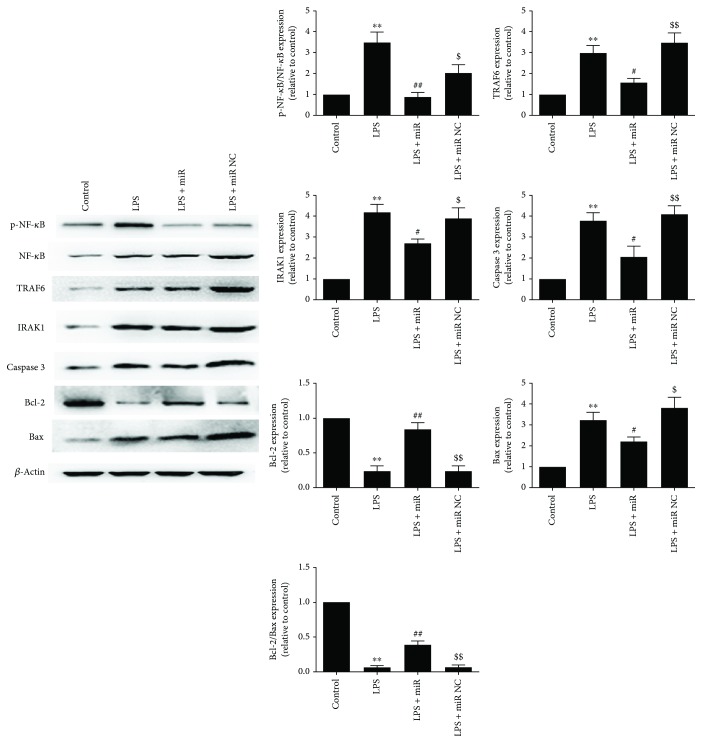
Overexpression of miR-146a downregulated TRAF6 and IRAK1 expression and suppressed NF-*κ*B activity in cardiomyocytes following LPS treatment. Representative images of the Western blot results are shown. The values were expressed as the mean ± SEM. ^∗∗^*P* < 0.01 versus the control group; ^#^*P* < 0.05 versus the LPS group; ^##^*P* < 0.01 versus the LPS group; ^$^*P* < 0.05 versus the LPS + miR group; ^$$^*P* < 0.01 versus the LPS + miR group.

According to the cell viability and ROS results, we further measured the expression of two important inflammatory-associated cytoplasmic protein kinases (TRAF6 and IRAK1) and proliferation/apoptosis proteins. As shown in Figure 5, LPS treatment significantly increased TRAF6 and IRAK1 expression and increased the phosphorylation level of NF-*κ*B which indicates the upregulated inflammatory status of cells. The miR-146a mimics significantly reduced the overexpression of TRAF6 and IRAK1 and also increased the Bcl-2/Bax ratio after LPS treatment; moreover, caspase 3 was significantly decreased in the miR-146a group which indicates the alleviated apoptosis status of cells.

### 3.3. Overexpression of miR-146a Alleviates Oxidative Injury Induced by LPS

**Figure 6:**
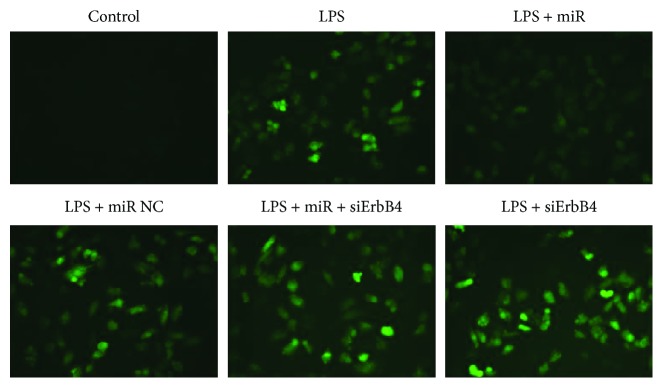
ROS level detected by DHE assay. Overexpression of miR-146a significantly reduced the ROS level post-LPS treatment, and siErbB4 significantly reversed the effect.

According to previous studies, we noticed that ErbB4 is a potential target of miR-146a; as a result, we investigate the role of ErbB4 in the effect of miR146a in LPS-treated H9C2 cells. Consequently, we increased miR-146a expression with miR-146a mimics and a negative control (NC). As shown in Figure 6, cells transfected with miR146a mimics showed significant decrease of ROS production compared to the LPS group and no significant difference was detected between the LPS group and the NC group. Then, with the addition of siErbB4, ROS production was significantly higher in the miR-146a + siErbB4 group than that in the miR-146a group. No significant difference was detected between the LPS and LPS + siErbB4 groups.

### 3.4. Overexpression of miR-146a Suppresses LPS-Induced Inflammatory Cytokine Production in Cardiomyocytes

**Figure 7:**
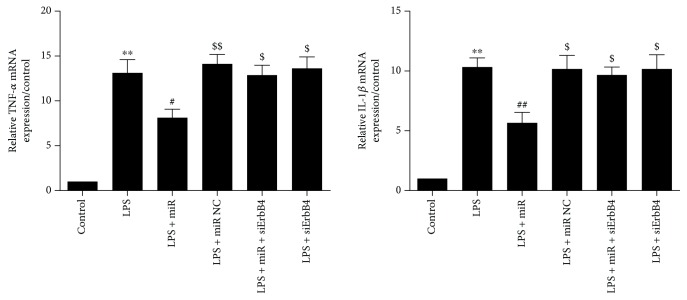
Overexpression of miR-146a decreased the levels of inflammatory cytokines in LPS-treated cardiomyocytes. The use of siErbB4 significantly reversed the effect of miR-146a. Data were expressed as mean ± SEM. ^∗∗^*P* < 0.01 versus the control group; ^#^*P* < 0.05 versus the LPS group; ^##^*P* < 0.01 versus the LPS group; ^$^*P* < 0.05 versus the LPS + miR group; ^$$^*P* < 0.01 versus the LPS + miR group.

In the miR-146a group, miR-146a mimics suppressed the mRNA expression of TNF-*α* and IL-1*β* compared with the control group (Figure 7). With the addition of siErbB4, the TNF-*α* and IL-1*β* mRNA expression was significantly increased. There are no significant differences between LPS + siErbB4 and LPS groups.

### 3.5. Overexpression of miR-146a Alleviates Cardiomyocyte Apoptosis Induced by LPS

**Figure 8:**
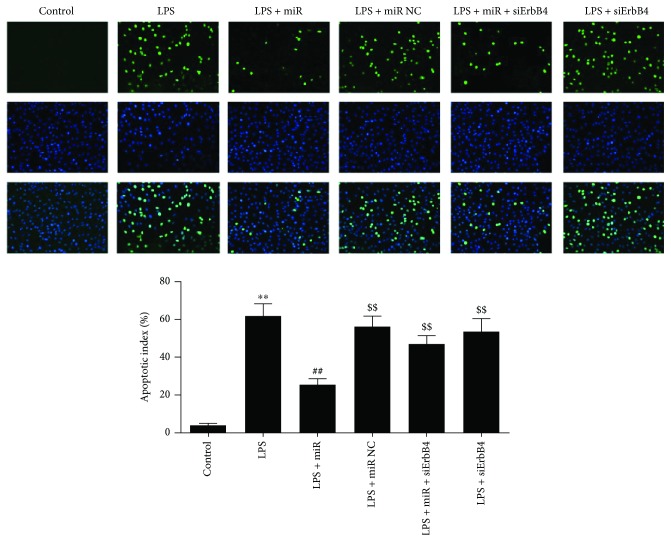
Evaluation of the apoptosis rate by TUNEL staining. Representative images of apoptosis are shown. The apoptotic cells were detected by TUNEL (green); the nuclei were detected by DAPI (blue). The results were expressed as the mean ± SEM. ^∗∗^*P* < 0.01 versus the control group; ^##^*P* < 0.01 versus the LPS group; ^$$^*P* < 0.01 versus the LPS + miR group.

As shown in Figure 8, the number of TUNEL-positive cells detected in cardiomyocytes was increased in the LPS group compared with the control group, indicating a relative higher apoptosis degree. There was a significant decrease in TUNEL-positive staining in the miR-146a group compared with the LPS group, demonstrating an antiapoptotic effect of miR-146a overexpression. siErbB4 suppressed the protective effect of miR-146a; the TUNEL-positive cells were significantly increased compared with the LPS + miR146a group. No significant difference was detected between the LPS group and the LPS + siErbB4 group.

### 3.6. Role of the ErbB4 in the Protective Effect of the Overexpression of miR-146a in LPS-Induced Cardiomyocyte Injury

**Figure 9:**
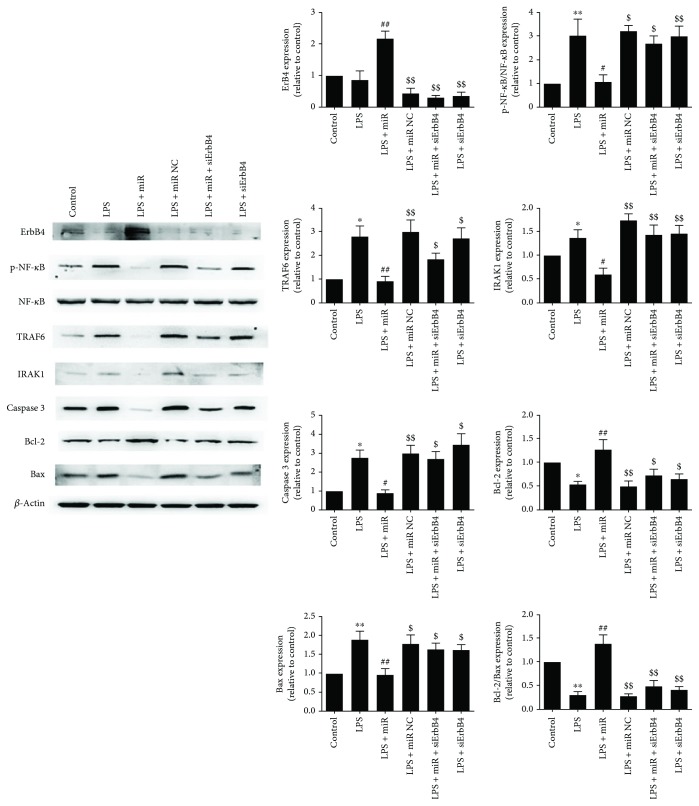
The effect of overexpression of miR-146a on the expression of ErbB4, TRAF6, IRAK1, NF-*κ*B, and caspase 3 activity and Bcl-2/Bax ratio. Representative images of the Western blot results are shown. The overexpression of miR-146a significantly increased the ErbB4 expression, decreased the expression of TRAF6, IRAK1, caspase 3, and the phosphorylation level of NF-*κ*B, and also increased the Bcl-2/Bax ratio; the effects were reversed by the use of siErbB4. The values were expressed as the mean ± SEM. ^∗^*P* < 0.05 versus the control group; ^∗∗^*P* < 0.01 versus the control group; ^#^*P* < 0.05 versus the LPS group; ^##^*P* < 0.01 versus the LPS group; ^$^*P* < 0.05 versus the LPS + miR group; ^$$^*P* < 0.01 versus the LPS + miR group.

To further investigate the protective effect of miR-146a in the LPS-induced cell injury, the protein levels of ErbB4, p-NF-*κ*B, NF-*κ*B, TRAF6, IRAK1, caspase 3, Bcl-2, and Bax were measured (*β*-actin as reference). As shown in Figure 9, miR-146a significantly increased the ErbB4 expression, decreased the expression of TRAF6, IRAK1, caspase 3, and the phosphorylation level of NF-*κ*B, and also increased the Bcl-2/Bax ratio compared with the LPS group. siErbB4 significantly reversed these effects in the LPS + miR + siErbB4 group. This suggested that ErbB4 is important for the protective effect of miR-146a overexpression against LPS-induced cardiomyocyte injury.

## 4. Discussion

In this study, a LPS-induced H9C2 cell sepsis model was established to investigate the function of miR-146a. First, the overexpression of miR146a significantly alleviated the cell injury induced by LPS treatment and improved the cell survival rate by decreasing proinflammatory cytokines and inhibiting apoptosis. Second, miR-146a protects cardiomyocytes from sepsis-induced cell dysfunction via inhibition of NF-*κ*B activity by targeting TRAF6 and IRAK1. Third, the protective effect of miR-146a reversed by the use of siErbB4 (ErbB4 is a potential binding target of miR-146a) demonstrated that miR-146a-mediated ErbB4 suppression is a potential causal mechanism of miR-146a protective effect in LPS-induced cardiac injury.

Lipopolysaccharide, a component of the outer membrane of Gram-negative bacteria that can cause systemic inflammatory response by entry lymphatic and circulatory systems, is widely used as an inducer of endotoxemia in scientific studies [8, 9]. LPS-induced sepsis is characterized by a systemic inflammatory process related to various cardiac diseases [10], and the underling mechanisms may include metabolic changes, autonomic dysregulation, mitochondrial dysfunction, cell apoptosis, and inflammation [11–15]. Previous studies suggested that TNF-*α* and IL-1*β* are involved in the initiation and regulation of inflammatory response [16]; the application of murine monoclonal anti-TNF antibodies induced a transient improvement in ventricular function in patients with sepsis [17]. And TNF-*α* also exerts a negative inotropic effect on the decrease of blood pressure and cardiac output [18]. Moreover, the ROS overproduction including superoxide, hydroxyl radical, and hydrogen peroxide also conduced to cellular damage [19]. In our study, the increased inflammatory cytokines induced by LPS were reflected by the overexpression of TNF-*α* and IL-1*β* compared with the control group. ROS production was significantly increased, and cell viability which reflects the cell condition post-LPS treatment was significantly decreased.

miR-146a has been proved to be a negative regulator of the NF-*κ*B activation pathway in innate immune responses and myocardial ischemia/reperfusion injury [20, 21]. And miR-146a can be induced by LPS via NF-*κ*B-dependent pathway [22]. Moreover, TRAF6 and IRAK1 are two key adapter molecules in the LPS/TLR4/NF-*κ*B pathway, identified as direct targets of miR-146a, and promote inflammation stimulated by proinflammatory cytokines including TNF-*α* and IL-1*β* [23]. Thomas et al. reported that IRAK1-deficient mice hearts showed resistance to LPS-induced contractile dysfunction through the repression of the NF-*κ*B pathway [24]. TRAF6 also play a crucial role in the induction of inflammatory responses. Chen demonstrated that it contains a RING domain that confers E3 ligase activity to induce autoubiquitination via catalyzation of lysine-63 (K63) polyubiquitination and TRAF6 suppression will reduce inflammatory responses mediated by NF-*κ*B and MAPK signaling pathways [25]. Consistent with these studies, miR-146 was significantly overproduced with the activation of NF-*κ*B in LPS-treated H9C2 cells and the transfection of miR-146a mimics significantly reduced NF-*κ*B activation as well as the expression of TRAF6 and IRAK1. Collectively, miR-146a in LPS/TLR4/NF-*κ*B signaling was regulated through a negative feedback loop, which can suppress acute inflammatory response.

To further identify the mechanism of miR-146a's effect, siErbB4 was used to investigate the relationship of miR-146a and ErbB4 in LPS-induced cardiomyocyte injury. NRG-1/ErbB signaling is widely known for its important role in cardiac and neuronal development. Ventricular muscle with the inactivation of ErbB4 may lead to severe dilated cardiomyopathy, and knockout of NRG-1 worsen the ventricular function post-Dox-induced cardiac injury [26, 27]. These studies showed the protective effect of NRG-1/ErbB signaling in both neonatal cardiac development and ventricular function postcardiotoxicity. Consistently, our results showed that miR-146a significantly increased the expression of ErbB4 and consequently showed the protective effect. We evaluated the apoptosis by TUNEL; with the application of miR-146a, the apoptotic index was significantly reduced compared with the LPS group, and siErbB4 abolished the protective effect of miR-146a in the LPS + miR-146a + siErbB4 group, revealing that the ErbB4 signaling is associated with the miR-146a's protection against LPS-induced cardiac injury. The expression of caspase 3, Bcl-2, and Bax also demonstrated the antiapoptotic effect of miR-146a and the ErbB4 signaling involvement in the LPS-induced cardiac injury. Moreover, the LPS-induced inflammatory cytokines were also decreased by the miR-146a and reversed by the application of siErbB4 which indicated that miR-146a in association with ErbB4 serve as a negative regulator of LPS-induced inflammatory cytokine production in cardiomyocytes. ROS production was also reversed by the use of siErbB4, indicating that the protective effect of miR-146a was closely related to the interaction between miR-146a and ErbB4. Takahiro Horie et al. reported that the inhibition of ErbB4 may be one of the reasons why those patients who receive concurrent Dox and trastuzumab therapy suffer from congestive heart failure (CHF). In their study, Dox treatment upregulated the expression of miR-146a which directly targets the ErbB4 3′UTR. Interestingly, they showed that the overexpression of miR-146a as well as that of siRNA against ErbB4 induced cell death in cardiomyocytes after Dox treatment. Controversially, in our study, miR-146a was also slightly increased post-LPS treatment; however, the exogenous overexpression of miR-146a significantly increased the ErbB4 expression and showed protective effect. From the present results, it is possible that the difference was caused by the treatment of Dox or LPS, the down- or upregulation of ErbB4 may be transient and need prolonged observation, and there may be a negative feedback loop to compensate for the ErbB4 expression variation that needs further investigation.

In summary, our study suggests that with the increased ErbB4 expression, the overexpression of miR-146a exhibits antioxidant activity, inhibits inflammation, and suppresses apoptotic effect, contributing to the attenuation of myocardial depression induced by LPS in H9C2 cells.

## Data Availability

The data used to support the findings of this study are available from the corresponding author upon request.
